# Absorption and translocation to the aerial part of magnetic carbon-coated nanoparticles through the root of different crop plants

**DOI:** 10.1186/1477-3155-8-26

**Published:** 2010-11-08

**Authors:** Zuny Cifuentes, Laura Custardoy, Jesús M de la Fuente, Clara Marquina, M Ricardo Ibarra, Diego Rubiales, Alejandro Pérez-de-Luque

**Affiliations:** 1 IFAPA, Centro Alameda del Obispo, Área de Mejora y Biotecnología, Avda. Menédez Pidal s/n, PO Box 3092, Córdoba, 14004 Spain; 2 Instituto de Ciencia de Materiales de Aragón (ICMA), CSIC-Universidad de Zaragoza, Pedro Cerbuna 12, Zaragoza, 50009 Spain; 3 Departamento de Física de la Materia Condensada, Universidad de Zaragoza, Pedro Cerbuna 12, Zaragoza, 50009 Spain; 4 Instituto de Nanociencia de Aragón, Universidad de Zaragoza, Campus Rio Ebro, Edificio i+d+i, Mariano Esquillor s/n. Zaragoza, 50018 Spain; 5 CSIC, Instituto de Agricultura Sostenible, Alameda del Obispo s/n, PO Box 4084, Córdoba, 14080 Spain

## Abstract

The development of nanodevices for agriculture and plant research will allow several new applications, ranging from treatments with agrochemicals to delivery of nucleic acids for genetic transformation. But a long way for research is still in front of us until such nanodevices could be widely used. Their behaviour inside the plants is not yet well known and the putative toxic effects for both, the plants directly exposed and/or the animals and humans, if the nanodevices reach the food chain, remain uncertain. In this work we show that magnetic carbon-coated nanoparticles forming a biocompatible magnetic fluid (bioferrofluid) can easily penetrate through the root in four different crop plants (pea, sunflower, tomato and wheat). They reach the vascular cylinder, move using the transpiration stream in the xylem vessels and spread through the aerial part of the plants in less than 24 hours. Accumulation of nanoparticles was detected in wheat leaf trichomes, suggesting a way for excretion/detoxification. This kind of studies is of great interest in order to unveil the movement and accumulation of nanoparticles in plant tissues for assessing further applications in the field or laboratory.

## Background

Several areas, such as medicine, materials science and electronics, have begun to benefit and apply nanotechnology for their research since some decades ago. However, only during the recent years, researchers from other disciplines start to see the potential applications of nanoscience, as it is the case of agriculture [1]. Nanosensors, smart delivery systems and nanomaterials (as for example, nanoparticles) appear as the most promising devices for application in agriculture and food industry. For example, using smart delivery systems in agriculture and plant research will open up new possibilities for multiple applications, from agrochemical treatments to genetic transformation [2,3]. However, it is not easy to adapt a technology developed for animals and humans to the plant kingdom. Effective means of nanoparticles application should be identified, and the behaviour, and their movement and accumulation within the plants should be understood.

During the last years, some works have been published about absorption and uptake of nanoparticles by plants, but mainly dealing with and focused on their putative adverse effects [4-8]. Nevertheless, in order to use nanoparticles as potential smart delivery systems, more systematic studies are needed to unveil the transport routes, the organs and tissues where nanoparticles tend to accumulate, and if there are differences regarding plant species and the kind of nanoparticles used. Such studies are important not only from the point of view of the application of nanoparticles in plants, but also for understanding putative toxic effects on plants and the possibilities of such nanodevices to accumulate in fruits and grains for further entry into the food chain.

In a previous research, we analyzed the penetration and transportation of magnetic carbon-coated nanoparticles through the leaves and aerial part of the plant in cucumber (*Cucurbita pepo*) [9,10]. The magnetic nature of our nanoparticles would allow further multiple applications once the nanoparticles are inside the plants. For example, the nanoparticles could be moved or immobilized in certain areas or tissues [9] applying a magnetic field, for delivering substances (drugs, DNA, etc.). In addition, they could work as contrast agents for magnetic resonance imaging (MRI) and be used for in vivo monitoring the movement and distribution of the nanoparticles (and their eventual load) inside the plant, enhancing such kind of studies [11]. Furthermore, hyperthermia [12] might be used for treatment of, for example, localized parts of trees affected by diseases or insect attacks. However, prior to the development of such applications a deep understanding on nanoparticle penetration and movement within the plant is needed.

In the present work, we have studied the absorption and translocation of magnetic carbon-coated nanoparticles through the root in four crop plants belonging to different families: sunflower (*Helianthus annuus*) from the family Compositae; tomato (*Lycopersicum sculentum*) from the Solanaceae; pea (*Pisum sativum*), from the Fabaceae; and wheat (*Triticum aestivum*), from the Triticeae.

## Methods

The same kind of carbon-coated iron nanoparticles used in previous studies [9,10] were produced in an arc-discharge furnace [13] based on the previously designed by Krätschmer-Huffman in 1990 [14]. Our arc-discharge furnace consist of a cylindrical chamber, in which there are two graphite electrodes: a stationary anode containing 10 μm diameter iron powders, and a moveable graphite cathode. An arc is produced between the graphite electrodes in a helium atmosphere. The graphite electrode is sublimed and builds up a powder deposit (soot) on the inner surface of the chamber. In this material we found: carbon nanostructures (as for example carbon nanotubes, amorphous carbon etc) and iron and iron oxides nanoparticles encapsulated in graphitic layers (leading to a particle- diameter size distribution centred at approximately 10 nm), together with a small amount of non-coated or partially coated metallic particles. These particles (which are not biocompatible) were eliminated by chemical etching, washing the soot with HCl 3M at 80°C. This procedure favours the formation of carboxylic groups on the graphitic shell, which, due to their hydrophobic nature, will contribute to the stability of the final particle suspension. In order to eliminate the amorphous carbon and therefore increase the concentration of magnetic nanoparticles, a magnetic purification of the powder is carried out. For this purpose, stable suspensions of the particles are prepared in a surfactant solution: 2.5 g of SDS in 500 ml of distilled water. A field gradient produced by 3KOe permanent magnet was used for magnetic separation of this suspension. The resultant powder was several times washed in water, before proceeding to nanoparticles suspension in manitol solution (1%) and further application.

**Figure 1:**
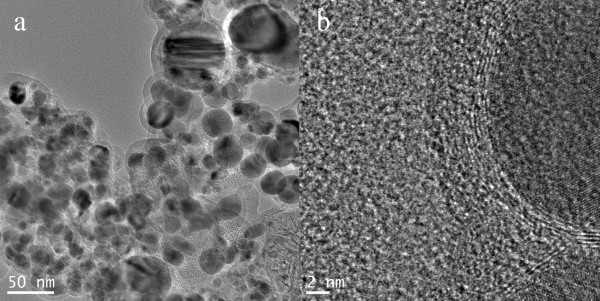
**TEM images at 300 kV using the cs image corrector (CEOS)**. a) Nanoparticles encapsulated in several layers of graphitic carbon, and surrounded by amorphous carbon. b) Detail showing the atomic planes of the nanoparticle metallic core.

HR-TEM images of the powder samples produced by arc discharge show spherical magnetic nanoparticles encapsulated in several layers of graphitic carbon, and surrounded by amorphous carbon (Figure 1a). HR-TEM also makes it possible to view the atomic planes of the nanoparticle metallic core (Figure 1b). The diameter of the particles has also been obtained. By analysing several images, the diameter probability distribution function can be obtained and plotted as a size distribution histogram. The powder sample produced by arc discharge contains particles of diameters ranging from 5 nm up to 50 nm, with the centre of the distribution at 10 nm. HR-TEM shows that after chemical etching the coating of the magnetic particles is complete. Hydrodynamic size was measured by Dynamic Light Scattering technique (Beckman Coulter N5 particle size analyser). The measurements showed that the carbon-coated magnetic particles in solution form aggregates ranging from 5 nm to several hundred nanometers, being the average hydrodynamic diameter 200 nm (See Additional file 1: Hydrodynamic size).

**Figure 2:**
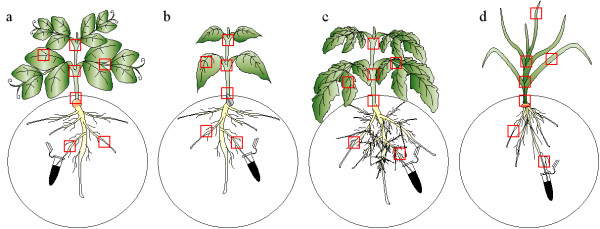
**Schematic representation of the Petri dish rizhotron with the four crops: a) pea; b) sunflower; c) tomato; d) wheat**. Squares indicates sampling points of plant tissues.

The plants were grown in vitro using a Petri dish system (rhizotron) allowing visualizing the roots [15]. When the plantlets developed the second pair of leaves in each species, nanoparticles [16,17] were applied to the roots as a suspension in manitol solution (1%) (Figure 2) immersing only some roots of each plantlet in the bioferrofluid. This allowed later to check if the nanoparticles could move to other roots. Samples of tissues from different parts of the plants (Figure 2) were taken after 24 and 48 hours and fixed for further microscopic analysis. Sections of samples were obtained either by using a vibratome or by hand cut, avoiding embedding and washing of nanoparticles from the tissues. Taking advantage of the black colour that present the bioferrofluid, a conventional light microscopy technique was used to follow its distribution, without observation of single nanoparticles or small aggregates, which requires electronic microscopy.

## Results and discussion

**Figure 3:**
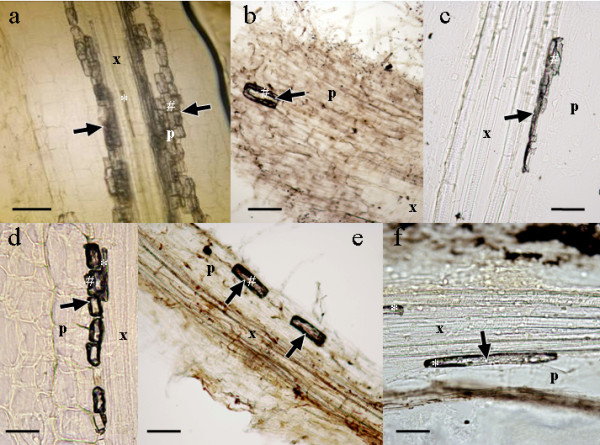
**Longitudinal sections of roots of pea (a, d), sunflower (b, e) and wheat (c, f)**. Arrows indicate accumulation of bioferrofluid in the cells. *, xylem containing ferrofluid; #, parenchimatic cell containing ferrofluid; p, parenchimatic cells; x, xylem vessels. Scale bars: a) and f), 50 μm; b) and e), 100 μm; c) and d), 25 μm.

Firstly we assessed that bioferrofluid was able to penetrate into the treated roots and to reach the vascular cylinder in a short period of time. Study of the samples taken at the point of application showed that after only 24 hours of exposure to the bioferrofluid, nanoparticles were able to leak into the vascular tissues of the tested crops (Figure 3). This indicates that application by immersing the roots into nanoparticle solutions is faster and more reliable in order to get big amounts of nanoparticles inside the plant, than applying the bioferrofluid through the leaves and aerial parts by pulverization or injection [9,10].

At this point, there are no studies about the real mechanism by which nanoparticles can penetrate into the plant cells. However, there is a recent work dealing with internalization of gold nanoparticles using tobacco protoplasts [18]. In such paper, the authors describe how gold nanoparticles penetrated into the protoplasts by endocytosis and were linked to different pathways upon their charge, including a clathrin-dependent pathway. So endocytosis appears as a reasonable way for internalization of nanoparticles. In fact, in a previous work [10] we found that internalized nanoparticles accumulate in clusters inside the cells, and despite cell membranes were not observed because the fixation method didn't preserve them, probably the nanoparticles are inside vesicles or cell organelles. In addition, the nanoparticles were suspended in mannitol, a solution more suitable for plants than gelafundin, and there are reports about enhancement of endocytosis by mannitol [19]. A recent paper [20] deals with penetration of gold nanoparticles through lipid membranes bypassing endocytosis. However, this entry way, although possible in the case of our carbon coated nanoparticles, is likely not common, because in such case a strong cytotoxicity (and probably phytotoxicity) should be observed.

Nanoparticles were detected easily in the xylem vessels of the four crops studied, but some differences were observed among species. Pea roots accumulated higher contents of bioferrofluid (Figure 3a) than sunflower or wheat, for example. This difference still remained after 48 hours of exposure to bioferrofluid (Figure 3d-f), suggesting that pea roots could be more permeable to nanoparticle penetration or that there is a lower transportation rate towards other plant parts, involving higher accumulation of nanoparticles at the application point.

**Figure 4:**
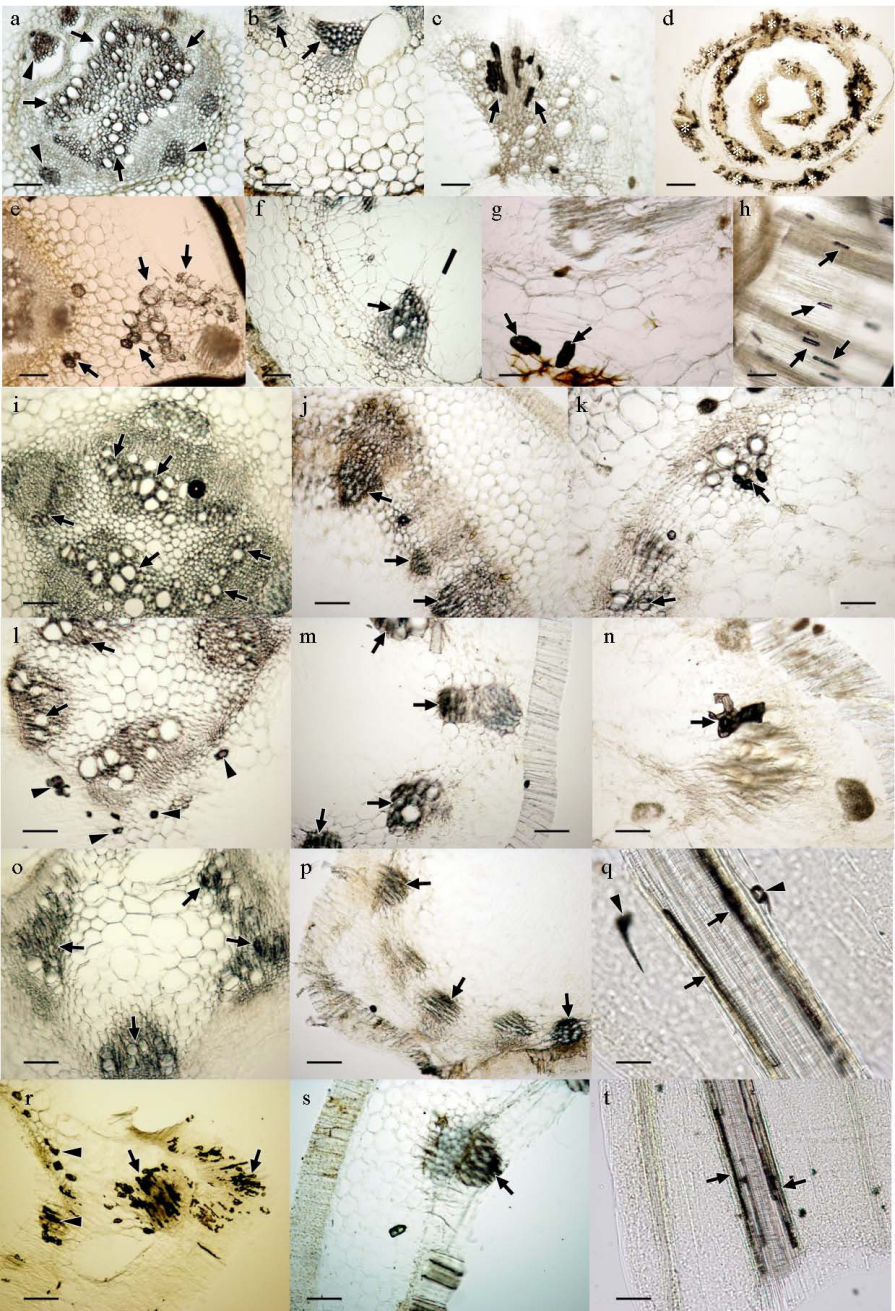
**Sections from different samples of the aerial parts of pea (a,e,i,l,o,r), sunflower (b,f,j,m,p,s), tomato (c,g,k,n) and wheat (d,h,q,t)**. a) Detail of the crown of pea after 24 h of exposure to bioferrofluid. b) *Idem *in sunflower. c) *Idem *in tomato. d) Crown of wheat after 24 h of exposure, showing an intense accumulation of bioferrofluid in tissues. e) Detail of the crown of pea after 48 h of exposure to bioferrofluid. f) *Idem *in sunflower. g) *Idem *in tomato. h) Detail of a longitudinal section in wheat after 48 h of exposure to bioferrofluid. i) Detail of a cross section of the first internode of pea after 24 h of exposure to bioferrofluid. j) *Idem *in sunflower. k) *Idem *in tomato. l) Detail of a cross section of the first internode of pea after 48 h of exposure to bioferrofluid. m) *Idem *in sunflower. n) *Idem *in tomato. o) Detail of a cross section of the second internode of pea after 24 h of exposure to bioferrofluid. p) *Idem *in sunflower. q) Detail of a longitudinal section of the second internode in wheat after 24 h of exposure to bioferrofluid. r) Detail of a cross section of the second internode of pea after 48 h of exposure to bioferrofluid. s) *Idem *in sunflower. t) Detail of a longitudinal section of the second internode in wheat after 48 h of exposure to bioferrofluid. Scale bars represent 100 μm, except in g), q) and t) whereas it represents 50 μm. Arrows indicate accumulation of nanoparticles in vascular tissues in a-c), f), i-t), and in cortical cells in e), g), h). Arrowheads indicate accumulation of nanoparticles in cortical cells in a), l), r), and in trichomes in q). Asterisks (*) indicate localization of vascular bundles.

After a successful uptake of the nanoparticles by the plant roots, we monitored the translocation of such nanoparticles into the aerial part. Figure 4a-h shows sections of the plant crown belonging to the four crop species after 24 and 48 hours of exposure to the bioferrofluid. The black deposit corresponding to the nanoparticles was clearly visible in the xylem vessels after 24 hours (Figure 4a-d). It implies that the nanoparticles had quickly moved towards the aerial part of the plants following the transpiration stream. Differential response among crop species was also noticed for nanoparticle translocation. Pea and wheat showed a high concentration of nanoparticles in the vascular tissues of the crown, whereas the presence of the bioferrofluid was less intense in tomato and sunflower. After 48 hours the nanoparticles were detected in cortical tissue from the crown of pea and wheat (Figure 4e, 4h) and even some cells in the cortex of tomato (Figure 4g), whereas no bioferrofluid was detected outside the vascular tissues of sunflower. This fact supports the idea that high amounts of nanoparticles penetrate quickly in the pea root and move into the aerial part, not being accumulated in the roots by a high transportation rate as suggested above. In the case of sunflower, it seems that the nanoparticles uptake through the roots is much slower than in the other species, and for that reason there is a lower accumulation after 24 hours of treatment. In addition, the bioferrofluid seems to be more restricted to the vascular tissues than in the other species.

**Figure 5:**
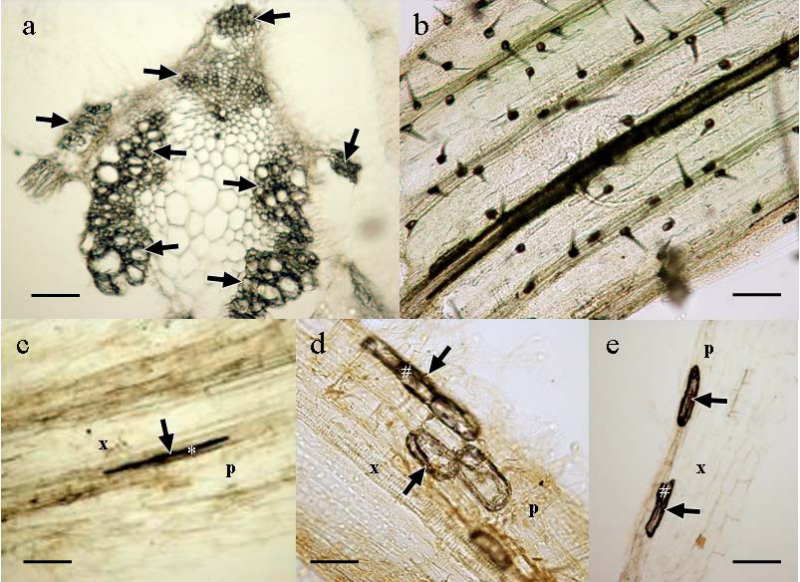
Sections from a pea petiole (a), wheat leaf (b), and pea (c), sunflower (d) and tomato (e) roots. a) Detail of a cross section of a petiole from the first internode of pea after 24 h of exposure to bioferrofluid. Arrows indicate accumulation of nanoparticles in vascular tissues. b) Detail of a longitudinal view of wheat leaf showing accumulation of bioferrofluid in trichomes. c) Detail of a longitudinal section of a root of pea not immersed into the bioferrofluid and after 48 h of exposure to bioferrofluid of opposite roots. Arrows indicate accumulation of nanoparticles in vascular tissues. d) *Idem *in sunflower. e) Idem in tomato. *, xylem containing ferrofluid; #, parenchimatic cell containing ferrofluid; p, parenchimatic cells; x, xylem vessels. Scale bars represent 100 μm, except in d) whereas it represents 50 μm.

Subsequent sections of upper parts of the plants confirmed that nanoparticles had spread and reached most of the aerial part after 24 hours of exposure to the bioferrofluid. Following the same pattern, accumulation of nanoparticles was detected in xylem vessels corresponding to the first (Figure 4i-k) and second (Figure 4o-q) internodes of the crops. Again, a higher presence of bioferrofluid was detected in pea and wheat compared with tomato and sunflower. However, such difference tends to disappear after 48 hours of exposure, showing an intense accumulation of nanoparticles in all the crops (Figure 4l-n, r-t). The bioferrofluid moved also towards the leaves and was detected in leaf petioles (Figure 5a).

It is remarkable that nanoparticles strongly accumulated in leaf trichomes of wheat plants (Figure 5b). The presence of nanoparticles in this kind of structures (trichomes) has been previously reported [10], but never in such a high amount, nor in the other crop species. Because trichomes can play a secretory function [21], it is possible that this accumulation of nanoparticles inside them indicates a putative detoxifying pathway in wheat. The reasons for the differences in accumulation of nanoparticles in trichomes are unclear, but we think that should be due to differences in the physiology of the plants: wheat belongs to the monocot group of plants, whereas the other three crops are dicots. It is known that different plant species show different behaviour regarding accumulation and excretion of heavy metals [22], so it is not surprising that such differences can also be found regarding metal nanoparticles.

Finally, the presence of nanoparticles in roots not exposed directly to the bioferrofluid was checked (Figure 5c-e). The characteristic black deposit was detected within the central cylinder of roots located diametrically opposite to the treated roots. These data suggest that nanoparticles had moved not only upwards through the xylem vessels following the transpiration stream, but also downwards, probably through the phloem and using the source-sink pressure gradient [23]. In fact, previous works have shown the translocation of nanoparticles applied on the aerial part of the plants into the roots [9], and there are evidences that radial transport from cell to cell occurs [10], which may involve the trafficking pathway to plasmodesmata. Once the nanoparticles are inside the cells, they can be transported via endosomes toward other areas and discharged outside the cells by exocytosis. In that case, Rab proteins should be involved in the process and direct the cargo to specific areas near plasmodesmata locations [24]. This mechanism allows transportation through the cell and would secure a pass through the endodermal cells, avoiding the Casparian strip. However, movement via apoplast of the nanoparticles is compatible with the previous mechanism, but the nanoparticles should enter the symplast way once they reach the endodermis and the Casparian strip.

Because these microscopic techniques allow observation only with low resolution, the bioferrofluid was usually visualized inside xylem vessels where big accumulations of nanoparticles took place. However, 48 hours after roots exposure to bioferrofluid, nanoparticles were also detected in vascular and cortical parenchimatic cells of the plants (Figure 4e, g, h, l, r). As stated above, this is also in accordance with previous reports about radial transport of carbon-coated magnetic nanoparticles between neighbouring cells [10], and indicates that radial transport allows the movement of nanoparticles outside the vascular tissues. Detailed studies using electronic microscopy are underway in order to unveil the nature of this transportation.

In summary, in this work we have presented results showing how carbon-coated magnetic nanoparticles can be absorbed by the root system of four different crop plants and spread using the vascular system to reach the whole plant. There are differences in the speed of absorption and distribution of the nanoparticles depending on the species, being faster in pea and wheat than in tomato and sunflower. In addition, it seems that sunflower shows a lower capability for radial movement of bioferrofluid outside the vascular tissues than the other crops. Within the first 24 hour of exposure to the suspension, the nanoparticles can reach the upper part of the plants, and in the case of wheat they accumulate inside leaf trichomes. After 48 hours of exposure, the bioferrofluid is located outside the vascular tissues (pea, tomato and wheat) and has moved downwards to non treated roots. This fast movement of the nanoparticles inside the plants can have an important impact for the development of nanoparticles as smart delivery systems inside the plant and further studies about their distribution and accumulation. It seems clear that root application is faster and more reliable than leaf treatments [9,10]. This might have implications in toxicity studies, because the way the nanoparticles are applied to the plants can strongly affect the final result. Further studies are needed to assess the effects of plant organs like flowers or fruits which tend to act as strong sink of plant resources (water and nutrients). There is a recent report [8] showing that fullerene nanoparticles can pass into the next generation of rice plants, which necessarily implies accumulation within the rice grains. Would that happen with bigger nanoparticles or nanomaterials synthesized with other components (i.e. starch, chitin, other metals...)?. In addition, despite the fact that plants could tolerate the presence of nanoparticles inside their tissues, an important question to be addressed is what happens with such nanoparticles if they move into the food chain. Could nanoparticles accumulated in a fruit/grain survive and pass through the digestive system of animals into the bloodstream?

## Competing interests

The authors declare that they have no competing interests.

## Authors' contributions

ZC carried out the nanoparticle treatments to the plants and the microscopy study, the processing of plant samples, and wrote the first manuscript draft. LC carried out the synthesis of nanoparticles and the bioferrofluid suspension. CM and MRI participated in the design of the nanoparticle synthesis and preparation of the suspension, in the design of the study and to the writing of parts of the manuscript. JMF contributed to the experimental design of nanoparticle synthesis and to the writing of parts of the manuscript. DR participated in the design of the study and helped in experiments of nanoparticle treatments to the plants. APL conceived the study, participated in the design and coordination of the work and helped to draft the manuscript. All authors read and approved the final manuscript.

## Supplementary Material

Additional file 1 **Hydrodynamic size**. The data show the hydrodynamic size of the nanoparticles measured by Dynamic Light Scattering technique. Click here for file
