# Supplementary material for: Absorption and translocation to the aerial part of magnetic carbon-coated nanoparticles through the root of different crop plants
Source: J Nanobiotechnology. 2010 Nov 8;8:26. doi: 10.1186/1477-3155-8-26 (PMC2994779; doi:10.1186/1477-3155-8-26)

Hydrodynamic size measured by Dynamic Light Scattering technique (Beckman Coulter N5 particle size analyser). The measurements showed that the carbon-coated magnetic particles in solution form aggregates ranging from 5 nm to several hundred nanometers, being the average hydrodynamic diameter 200 nm.

Unimodal Results Summary

Rept#.	Mean (nm)	P.I.	Diff.Coef (m^2/s)	Counts/s	Baseline Error	Overflow
Rept.1	203.4	0.438	2.41e-12	3.62e+05	2.65%	0
Rept.2	209.2	0.508	2.34e-12	3.20e+05	1.31%	0
Rept.3	228.1	0.661	2.15e-12	3.31e+05	4.37%	0
Average	213.6 ± 10.54	0.536 ± 0.093				

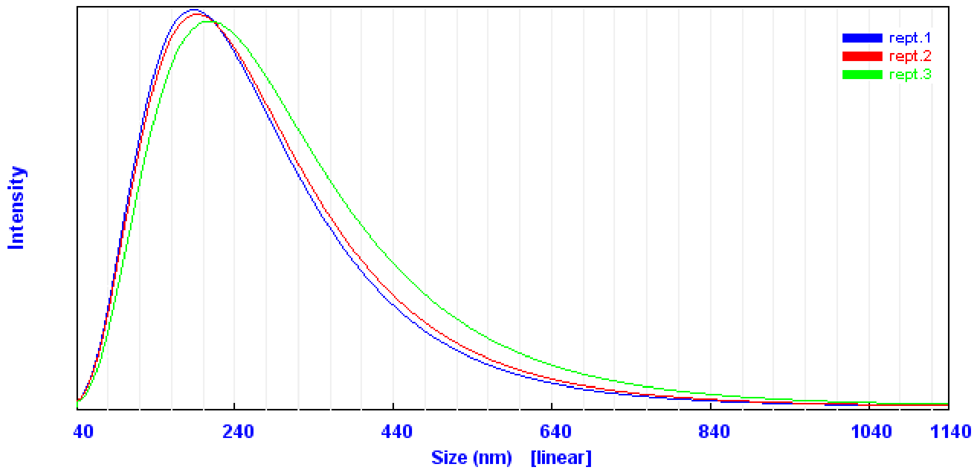

Supplement: Additional file 1 — Hydrodynamic size. The data show the hydrodynamic size of the nanoparticles measured by Dynamic Light Scattering technique. [file 1477-3155-8-26-S1.PDF]
